# Neurocognitive bases of self-monitoring of inner speech in hallucination prone individuals

**DOI:** 10.1038/s41598-023-32042-4

**Published:** 2023-04-17

**Authors:** Christian Stephan-Otto, Christian Núñez, Federica Lombardini, Maria Rosa Cambra-Martí, Susana Ochoa, Carl Senior, Gildas Brébion

**Affiliations:** 1 Institut de Recerca Sant Joan de Déu, Esplugues de Llobregat, Spain; 2 Parc Sanitari Sant Joan de Déu, Sant Boi de Llobregat, Spain; 3 Centro de Investigación Biomédica en Red de Salud Mental (CIBERSAM), Madrid, Spain; 4 School of Life & Health Sciences, Aston University, Birmingham, UK; 5 University of Gibraltar, Gibraltar, UK

**Keywords:** Language, Disorders of consciousness, Neural circuits, Human behaviour

## Abstract

Verbal hallucinations in schizophrenia patients might be seen as internal verbal productions mistaken for perceptions as a result of over-salient inner speech and/or defective self-monitoring processes. Similar cognitive mechanisms might underpin verbal hallucination proneness in the general population. We investigated, in a non-clinical sample, the cerebral activity associated with verbal hallucinatory predisposition during false recognition of familiar words —assumed to stem from poor monitoring of inner speech—vs. uncommon words. Thirty-seven healthy participants underwent a verbal recognition task. High- and low-frequency words were presented outside the scanner. In the scanner, the participants were then required to recognize the target words among equivalent distractors. Results showed that verbal hallucination proneness was associated with higher rates of false recognition of high-frequency words. It was further associated with activation of language and decisional brain areas during false recognitions of low-, but not high-, frequency words, and with activation of a recollective brain area during correct recognitions of low-, but not high-, frequency words. The increased tendency to report familiar words as targets, along with a lack of activation of the language, recollective, and decisional brain areas necessary for their judgement, suggests failure in the self-monitoring of inner speech in verbal hallucination-prone individuals.

## Introduction

Hallucinations, which are a hallmark of schizophrenia, also occur with a significant incidence in the general population^1–3^, although their prevalence rate varies widely across studies. Certain cognitive mechanisms have been demonstrated to underlie both hallucinations in non-clinical individuals and those experienced by patients with schizophrenia, suggesting a continuum from normality to pathological experience^4–7^. One of the shared cognitive bases of non-clinical and clinical hallucinations appears to be dysfunction in source monitoring^8–10^.

Source monitoring is a broad concept which encompasses various overlapping functions such as reality monitoring/discrimination—the ability to distinguish imagined from perceived events—, self monitoring—the ability to recognize one’s overt or covert productions as one’s own—, and source memory—the ability to remember, rather than identify, the origin of information. Impairment in various types of source-monitoring processes in individuals with hallucinations has been studied through a plurality of paradigms. Notably, signal detection tasks have been used to study reality-discrimination processes. It has been repeatedly observed that a liberal response bias, reflecting a tendency to make false detections of auditory signals that were not emitted, is related to hallucinations in schizophrenia patients^11–13^ and to hallucination proneness in non-clinical individuals^11,14–19^. Auditory verbal imagery^20^ and prior expectations^21,22^ have been demonstrated to play a crucial role in the false detection of speech in hallucination-prone individuals. Impairments in source memory have been demonstrated by altered response bias in recognition tasks. In such tasks participants are not required to detect stimuli but rather to remember after a delay whether stimuli have been previously presented. Hallucinations in schizophrenia patients have been found to be associated with a liberal response bias in word recognition^23–25^ and picture recognition^26,27^, reflecting a tendency to falsely remember words or pictures which had not been presented in the encoding phase. A liberal response bias in word recognition^25,28^ and picture recognition^27^ was also found to be associated with hallucination proneness in non-clinical individuals**.** Two studies used the Deese–Roediger–McDermott paradigm, which induces false recognition of words, to investigate hallucination proneness in healthy participants. The authors did not calculate a response bias but did report that auditory hallucination proneness was correlated with increased rates of false recognitions of words strongly associated with target words^29^ and of non-associated words with negative emotional valence^30^.

Like false detections, false recognitions may be seen as stemming from self-monitoring failure, with inability to discriminate internally-generated from externally-produced stimuli. Indeed, non-target stimuli in the recognition test may seem familiar because of shared features with internal representations of words or images, and therefore they are mistaken for stimuli presented at encoding. As far as verbal hallucinations are concerned, false recognitions of non-target words might result from the mistaking of inner verbal productions for verbal experimental material. In line with this, a recent study in which we contrasted high- and low-frequency words revealed that verbal hallucinations in patients, as well as hallucination proneness in healthy individuals, were specifically related to the false recognition of high-frequency words^25^. In a recognition test, individuals presenting verbal hallucinations might fail to distinguish the words previously presented in the experimental target list from those that seem familiar on account of their readily available internal representation through inner speech, as might be the case with the high-frequency words. According to the source-monitoring framework, confusion between imagination and perception may arise from dysfunctional judgment —i.e., internal/external comparison—processes, or from the fact that internal events either present the characteristics of perceived events or lack those of imagined events^8^. Thus, such failure in the self-monitoring of verbal material might stem from an altered threshold in evaluation/decision processes, i.e., a tendency to laxly give the status of perception to internally-produced verbal events. Alternatively, independent of the integrity of the internal/external comparison processes, inner verbal productions might be abnormally salient and thereby less distinguishable from externally-presented words.

Brain studies have demonstrated across a variety of paradigms that verbal hallucinations are associated with abnormal activity and lateralization of temporal auditory regions, and with altered functional connectivity within the language network^31–33^. While dysfunction of the auditory and language processing regions might generally underlie voice hearing, similarities and differences in neural alterations between clinical and non-clinical hallucinations, and between state and trait hallucinations, are not clearly delineated. According to a recent review, decreased asymmetry seems to be observed also in non-clinical voice hearers; activity of the left superior temporal gyrus might differentiate state vs- trait verbal hallucinations^31^. Studies that have examined the cortical underpinnings of reality-monitoring/self-monitoring processes suggest that verbal hallucinations are further associated with dysfunction in various regions involved in the appraisal and monitoring of self-generated information^34–37^. These regions include the anterior cingulate cortex as well as subregions of the temporal lobe—involved in recollective experience and feeling of familiarity—and anterior prefrontal cortex.

In this neuroimaging study of a non-clinical sample, we aimed to determine whether the previously observed role of word frequency in the association between false recognitions and verbal hallucinations^25^ corresponded to a differential cortical processing, which would enhance the view of an implication of inner speech in this symptom. False recognitions are known to activate recollective brain regions largely overlapping with those activated by correct recognitions, notably in the parietal and temporal cortex, while prefrontal regions supporting monitoring and cognitive control processes have been found to be more active during false than correct recognitions^38^. FMRI studies have revealed that low-frequency words elicit greater activation of the left inferior frontal gyrus and other language-related brain regions than do high-frequency words, allegedly reflecting the fact that high-frequency words are more easily accessed and require less phonological and semantic processing^39^. We contrasted high- and low-frequency words and hypothesized that verbal hallucination proneness was associated with increased rates of false recognitions of high-frequency words. Verbal hallucination proneness was further expected to be associated with activation of language-related regions, as well as regions involved in recollection and decision processes, during false recognitions of non-presented words. We investigated whether the putative specific association with false recognitions of high-frequency words corresponds to a distinct cerebral activation pattern for the high-frequency words when compared to those of low-frequency. In hallucination-prone participants, false recognitions of high-frequency words might be associated with abnormal activation of decisional brain regions, which would suggest altered self-monitoring processes. On the other hand, the high-frequency words might undergo an atypical processing at the language level, suggesting abnormality of inner speech.

In order to demonstrate that impairment in the monitoring of inner speech is a specific underpinning of verbal hallucinations rather than a general feature of psychosis, we also studied potential associations of response bias and cerebral activity with two factors similarly involved in psychotic experience, namely visual imagery and delusion proneness. No association with increased rates of false recognitions or increased activity in language brain regions was expected for either of them. Potential associations with activity in other brain areas, such as visual, recollective, and decisional areas, were explored. With respect to word frequency effects, visual imagery was expected to impact the cortical processing of high-frequency words, which were concrete and lent themselves readily to the formation of visual mental images, in contrast to uncommon words. The cortical impact of delusion proneness was not expected to be modulated by word frequency.

## Method

### Participants

Thirty-seven (18 female) Spanish-speaking participants with normal or corrected-to-normal vision were recruited from the general population by means of announcements: age: mean = 38.8, sd = 11.3; education level: median = 5 [1 = no studies; 2 = uncompleted primary studies; 3 = completed primary studies; 4 = high school uncompleted; 5 = high school completed; 6 = undergraduate studies; 7 = bachelor’s or master’s degree; 8 = doctorate]; verbal IQ (Word Accentuation test^40^: mean = 103.1, sd = 7.8. The inclusion criteria were age between 18 and 60 years and fluency in Spanish. The exclusion criteria were neurological or mental illness, intellectual disability, head injury, alcohol or drug abuse in the past six months, and current severe physical disease, as well as the standard exclusion criteria for participation in fMRI procedures, namely claustrophobia and metallic implants including fitted pacemakers and cochlear implants. The study was approved by the ethics committee of the Parc Sanitari Sant Joan de Déu, Barcelona, Spain, and it was conducted in accordance with the guidelines and regulations relevant for experimental studies of human subjects. All participants provided written informed consent before the task was administered.

### Scales for hallucination proneness, delusion proneness, and visual imagery

Hallucination proneness was assessed by means of a Spanish adaptation of the Launay-Slade Hallucination Scale (LSHS^41,42^), a self-questionnaire which measures proneness to hallucinatory experiences in various modalities. Two additional items were mixed with the LSHS items, although they were not taken into account in the computation of the LSHS score: ‘*I can easily identify animals or things in the clouds’*, and ‘*When I see spots (of painting, humidity…), I can see faces, silhouettes, or objects in them’*. Similar to the LSHS items, each of these new items had to be rated from 0 to 3 by the participants according to the frequency of the experience. The total score obtained on these two items constituted a visual imagery score (m = 2.16, sd = 1.38; range: 0–5). This visual imagery subscale, meant to assess the spontaneity and abundance of visual imagery rather than its vividness, had been validated and already used in a previous study of healthy participants^43^. A global hallucination proneness score was tallied by adding up the sub-scores for all LSHS items excluding the two new items (m = 6.73, sd = 5.19; range: 0–19). In addition, a verbal hallucination proneness score was computed by adding up the sub-scores obtained on the corresponding items (‘*In the past, I have had the experience of hearing a person’s voice and then found that no one was there*’, ‘*I often hear a voice speaking my thoughts aloud*’, and ‘*I have been troubled by hearing voices in my head’* (m = 0.73, sd = 1.19; range: 0–4). Proneness to delusions was assessed by means of the Peters Delusion Inventory scale^44^ (m = 7.9, sd = 7.6; range: 0–33).

The verbal hallucination and delusion proneness scores did not follow normal distribution; they were normalised by square root transformation before data analysis.

### Material

Six lists of 24 concrete nouns, equivalent in the total number of syllables but differing in the word frequency of use (*Corpus de Referencia del Español Actual*), were constructed. Three lists included high-frequency words (e.g., *book*, *dress*) (average frequency per million: m = 123.5, m = 122.9, and m = 120.3, respectively) and 3 included low-frequency words (e.g., *apron*, *whistle*) (average frequency per million: m = 2.96 for each list). Two high-frequency and 2 low-frequency lists were used as targets and the remaining two lists (1 high- and 1 low-frequency) were used as distractors. Two of the target lists (1 high- and 1 low-frequency), referred to as ‘read’ lists, were to be read by the participant, and the other two (1 high- and 1 low-frequency), referred to as ‘heard’ lists, were to be read aloud by the experimenter. Each target list was assigned to the ‘read’ or ‘heard’ condition in a counterbalanced way. The order of the conditions (*heard-read-heard-read* or *read-heard-read-heard*) and type of list (*high-high-low-low*, or *low-low–high-high*) was counterbalanced as well.

### Procedure

The scales and fMRI task were administered in one session of approximately 2 h. The participants received financial compensation.

## Outside scanner

The four target lists (1 high-frequency/heard, 1 high-frequency/read, 1 low-frequency/heard, 1 low-frequency/read), each displayed on a sheet of paper, were presented and the participants were instructed to memorize the words. In the ‘read’ condition they were required to read the word list aloud once, while in the ‘heard’ condition the experimenter read the word list aloud once. In order to prevent a floor effect, we split the lists into two half-lists. After the reading or hearing of each half-list (12 words), the participants were required to write down as many words as they could remember.

## Inside scanner

*Read/heard discrimination task:* The 96 target words were presented on the screen, one by one and in random order. The participants had to press one of two keys to indicate, after each word, whether it had been ‘read’ or ‘heard’ [The results of this task will not be reported on in this paper].

*Old/new recognition task*: Forty-eight target words (12 from each target list) and 48 distractors (24 high- and 24 low-frequency words) were presented in pseudo-random order, one by one for 3.5 s, separated by fixation crosses with random durations between 5.5 and 9 s extracted from an exponential distribution, with mean = 6.68 s. The participants had to press one of two keys to indicate, after each word, whether it had been previously presented in the target lists or was new.

Before the task began, two short lists of words (1 ‘heard’ and 1 ‘read’) were presented as practice outside the scanner. In the scanner, a few practice trials were also administered.

### fMRI data acquisition

MRI data for the participants were acquired with a General Electric 1.5 Tesla Signa HDe scanner (General Electric Healthcare, Milwaukee, WI, USA) at Parc Sanitari Sant Joan de Déu, using an 8-channel head coil. For each participant, a high-resolution T1-weighted FSPGR structural image with the axial plane parallel to the AC-PC axis was acquired using the following parameters: 2 mm slice thickness, TR = 12.24 ms, TE = 3.84 ms, FOV = 24 cm, acquisition matrix = 512 × 512, flip angle = 20°, voxel size = 0.47 × 0.47 × 2.00 mm^3^. A T2*-weighted functional echoplanar imaging sequence depicting BOLD contrast was also obtained. In total, 294 volumes were collected with AC-PC axial orientation, with the following scanning parameters: 26 slices, 4 mm thickness, 1 mm gap, TR = 2000 ms, TE = 40 ms, FOV = 24 cm, acquisition matrix = 64 × 64, flip angle = 90°, voxel size = 3.75 × 3.75 × 5.00 mm^3^. The first 7 volumes in each run were discarded to allow for magnetic saturation effects. Visual stimuli were presented on a rear projection screen and viewed through a mirror mounted on the head coil, and all responses were collected with an MR-compatible response box (fORP, Current Designs, Inc., USA; www.curdes.com).

### fMRI data preprocessing

Imaging data were analyzed using SPM12 (Wellcome Department of Imaging Neuroscience, London; www.fil.ion.ucl.ac.uk/spm) running under MATLAB (Release 2009a, The MathWorks, Inc., Natick, Massachusetts). All of the functional volumes for each participant were spatially realigned to the mean image in each series, in order to correct for small head movements. Motion parameters were examined for each subject to ensure that no movements larger than the voxel size were present. The resulting series were warped into MNI space using isotropic voxels (3 × 3 × 3 mm^3^) with SPM’s standard normalization procedure, and then spatially smoothed using a Gaussian kernel of 8 mm full-width-at-half-maximum.

### fMRI data analysis

The preprocessed fMRI data were analyzed with an event-related model, using SPM12. In order to assess random effects at the individual level, the activity associated with the experimental conditions was modelled with a hemodynamic response function (HRF) and its time derivative. Displacement and rotation motion parameters were included as confounds in the individual model. A 200s high-pass filter cut-off was used to remove low frequency noise, together with a first-order autoregression model to correct for temporal autocorrelation.

Four event types were determined by the responses in the old/new recognition task: target words identified as old (correct recognitions), distractors identified as new (correct rejections), target words erroneously identified as new (omissions), and distractors erroneously identified as old (false recognitions). Linear contrasts were constructed to test the experimental effects of interest. These contrasts were entered into a second level analysis in which subjects were treated as a random effect.

The resulting statistical parametric maps were generated using a cluster-defining threshold at voxel level defined by *p* < 0.001 and a cluster-level threshold defined by a family-wise-error (FWE) corrected *p* < 0.05. When necessary, a more restrictive FWE-corrected threshold at voxel level was used to separate brain activity clusters that extended across several brain structures.

### Measures

The numbers of correctly recalled high- and low-frequency words in the free recall task were tallied. The numbers of high- and low-frequency target words correctly reported as targets in the recognition task, as well as the numbers of high- and low-frequency distractor words erroneously reported as targets (false recognitions), were recorded with the computer, as were the response times for each type of response. The numbers of correctly and erroneously reported words were combined to compute a recognition efficiency index, Pr, reflecting the ability to discriminate target words from distractors (rate of correct recognitions minus rate of false recognitions), and a response bias index, Br, reflecting the tendency to report distractor words as targets (rate of false recognitions/1-Pr)^45^. Pr and Br indices were computed for each type of word (high frequency, low frequency), and an averaged Pr index was derived (Pr-global).

### Statistical design

#### Behavioural data

First, the word frequency effects were tested by contrasting the numbers of correctly recalled high- vs. low-frequency words, as well as the Pr indices for high- vs. low-frequency words (t-tests).

Regression analyses were then conducted on the response bias for high- and low-frequency words, and on the response times for the false recognitions of high- and low-frequency words. A regression analysis was computed for each variable, with the rating scale scores (LSHS, delusion proneness, and visual imagery) and four socio-demographic measures (age, sex, education level, and verbal IQ) as predictors. These latter measures were entered to control for their potentially confounding effect on the investigated associations, as sociodemographic factors have been demonstrated to have an impact on verbal memory^46,47^. In the event that a significant association with the LSHS score was observed, a post-hoc analysis of the effect of hallucination proneness was conducted. The regression analysis of the variable was recomputed after replacing the LSHS score with the verbal hallucination proneness score to test the hypothesis that verbal hallucinations were specifically involved in false recognitions.

#### Neuroimaging data

FMRI analyses were conducted on the correctly recognized high- and low-frequency target words and on the false recognitions of high- and low-frequency distractor words. In a first set of analyses, the verbal hallucination proneness score was entered as covariate along with the visual imagery score to determine the effect of each while controlling for their potential overlap. The Pr-global index was also entered in the model to control for the impact of recognition efficiency. Lastly, only sex and verbal IQ—which were found to impact cerebral activity in our previous studies—were entered to reduce the number of covariates. Preliminary analyses of the potential effect of each socio-demographic variable did not reveal any association of age or education level with cerebral activity in any of the contrasts studied. Then, two other sets of analyses were conducted on the same contrasts to determine the specificity of the cerebral activity associated with verbal hallucination proneness. The verbal hallucination proneness score in the model was replaced first by the LSHS score and then by the delusion proneness score, while the same other covariates were used.

## Results

### Behavioural data

T-tests revealed that the participants recalled significantly more high- than low-frequency words (m = 23.3, sd = 5.2 vs. m = 19.7, sd = 3.2; t(36) = 5.98, *p* < 0.0001). On the other hand, they demonstrated greater recognition of the low- than of the high-frequency words (m = 0.56, sd = 0.17 vs. m = 0.51, sd = 0.18; t(36) = 2.56, *p* < 0.015).

#### Br-high frequency

Regression analysis indicated that both visual imagery and delusion proneness scores made a near-zero contribution to the response bias, and so they were removed from the predictors. A regression analysis involving only LSHS score and the socio-demographic measures revealed that the LSHS score was a significant predictor of Br-high frequency, in the sense that global hallucination proneness was associated with an increased tendency to make false recognitions of non-presented high-frequency words, as expected (β = 0.42, *p* < 0.05). The post-hoc analysis indicated that, when the verbal hallucination proneness subscore was entered in this model instead of the LSHS score, it also made a significant contribution to Br-high frequency (β = 0.38, *p* < 0.05). Education level made a trend contribution to Br-high frequency in this latter model (β = 0.48, *p* < 0.09), while no significant association emerged for age (β = 0.42, *p* > 0.10), sex (β = 0.09, *p* > 0.10), or verbal IQ (β = − 0.25, *p* > 0.10).

#### Br-low frequency

The LSHS score was strongly associated with the response bias for the low-frequency words (β = 0.74, *p* < 0.015) while neither visual imagery (β = − 0.41) nor delusion proneness (β = − 0.21) score made any significant contribution to it (*p* > 0.10 in both cases). When the verbal hallucination proneness score was entered in the model instead of the LSHS score in the post-hoc analysis, its contribution to the response bias did not reach statistical significance (β = 0.41, *p* > 0.10), and no significant association was observed for visual imagery (β = − 0.21), delusion proneness (β = − 0.18), age (β = − 0.12), sex (β = 0.27), education level (β = 0.11), or verbal IQ (β = 0.17) (*p* > 0.10 in all cases).

#### Response time for the false recognitions of high-frequency words

The LSHS score did not make any contribution to the response time (β = − 0.05, *p* > 0.84) while the delusion proneness score was negatively associated with it (β = − 0.66, *p* < 0.01). No significant association with the visual imagery score was observed (β = 0.14). Education level made a significant (β = − 0.68, *p* < 0.05) and age a trend (β = − 0.49, *p* < 0.08) contribution to the response time, while sex (β = 0.07) and verbal IQ (β = 0.04) were unrelated to it.

#### Response time for the false recognitions of low-frequency words

No association with the LSHS score was observed (β = − 0.17, *p* > 0.52). The delusion proneness score was again negatively associated with the response time (β = − 0.56, *p* < 0.025), while the visual imagery score was positively associated with it (β = 0.56, *p* < 0.05). Education level tended to make a contribution to the response time (β = − 0.57, *p* < 0.06), while age (β = − 0.32), sex (β = 0.16), and verbal IQ (β = − 0.25) were not significantly associated with it (*p* > 0.10 in all cases).

### Neuroimaging data

**Figure 1:**
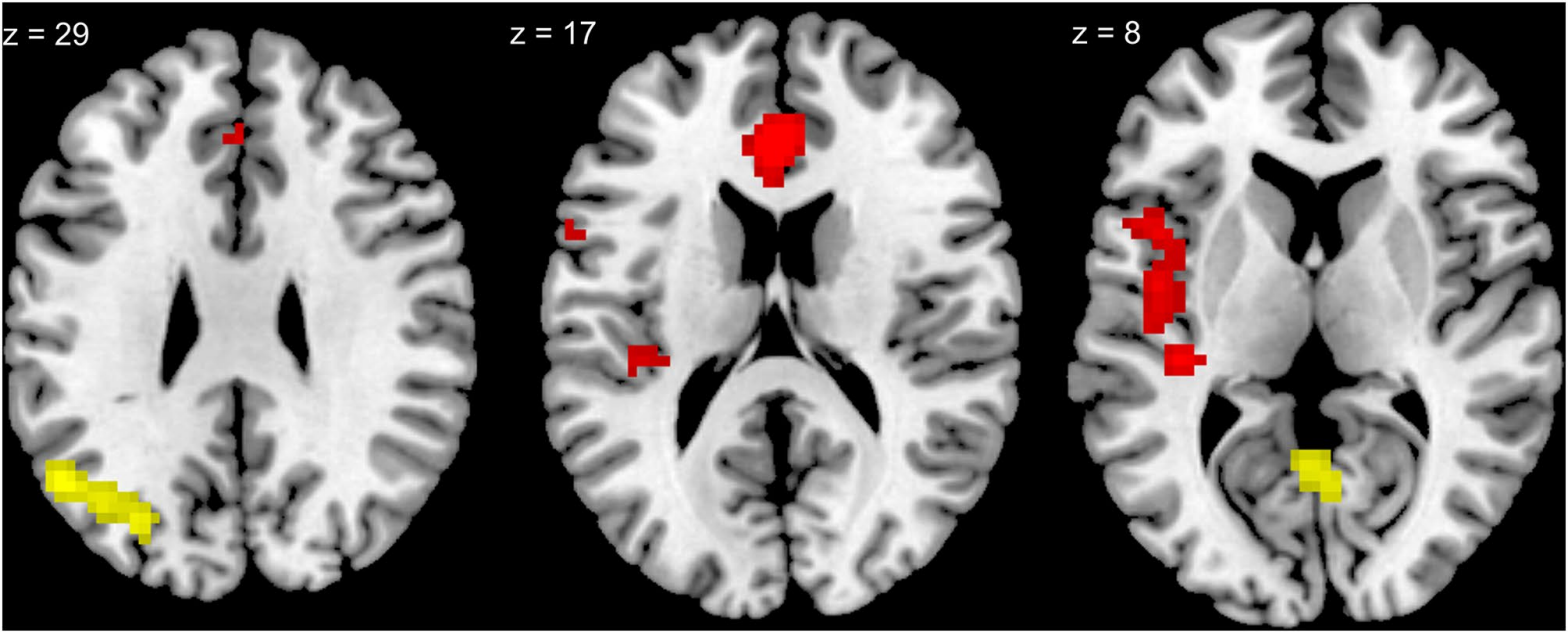
Activation clusters positively associated with verbal hallucination proneness score during correct recognitions (yellow) and false recognitions (red) of low-frequency words, while controlling for visual imagery score, sex, verbal IQ, and Pr-global. The slices depicting MNI coordinates derive from peak activations for the low-frequency word contrasts reported in Tables 1 and 2.

**Figure 2:**
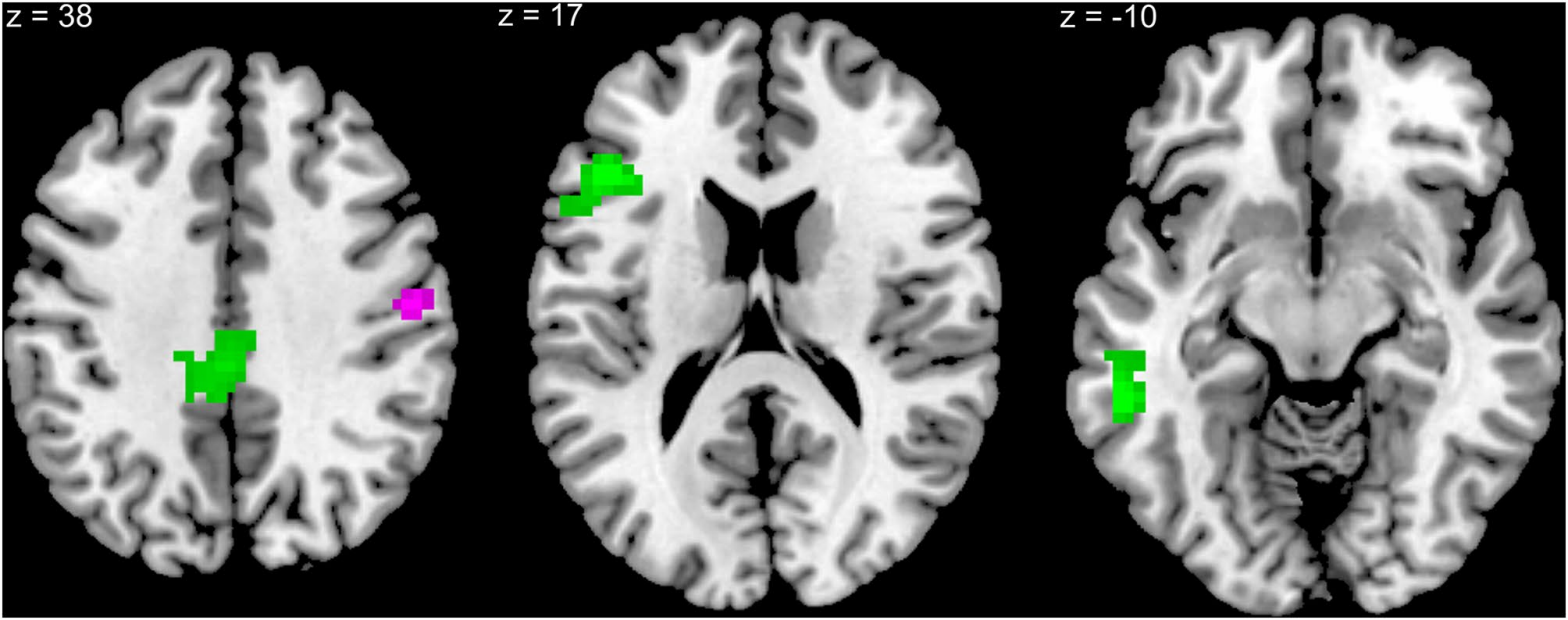
Activation clusters negatively associated with visual imagery score during correct recognitions (purple) and false recognitions (green) of high-frequency words, while controlling for verbal hallucination proneness score, sex, verbal IQ, and Pr-global. The slices depicting MNI coordinates derive from peak activations for the high-frequency word contrasts reported in Tables 1 and 2.

The results of the analyses conducted with the verbal hallucination proneness score as covariate are reported in Tables 1 and 2. Verbal hallucination proneness was significantly associated with activation of language areas (left Heschl’s gyrus, Broca’s area) and of the anterior cingulate during false recognitions of non-target words, as expected, while an association with activation of a recollective area, the left angular gyrus, emerged during correct recognitions of target words. However, these associations with correct and false recognitions were observed only for the low-frequency words (see Fig. 1). With respect to visual imagery score, associations with decreased activation of various brain areas were observed, and they pertained to the correct and false recognitions of high-frequency, but not low-frequency words, as expected (see Fig. 2). In particular, the left planum temporale and Broca’s area were under-activated during the false recognitions of these words, as were the posterior cingulate and right cerebellum (crus 1).

**Table 1:** Brain activation areas significantly associated with each covariate (verbal hallucination proneness, visual imagery, sex, verbal IQ, and Pr-global) during the correct recognition of high- and low-frequency words in the 37 participants.

Contrasts	Covariates	Cluster size and significance (p_FWE_)	Cluster peak coordinates (MNI)	Region
Correct recognition of high-frequency words	Verbal hallucination	–	–	
	Visual imagery	Neg 90 (0.021)	54,-13,38	Right postcentral gyrus
	sex	–	–	
	Verbal IQ	–	–	
	Pr-global	–	–	
Correct recognition of low-frequency words	Verbal hallucination	Pos. 122 (0.005)	-51, -61, 29	Left angular +
			-30, -73, 32	Left middle occipital
		Pos. 96 (0.015)	6, -64, 5	Lingual
	Visual imagery	–	–	
	Sex	–	–	
	Verbal IQ	–	–	
	Pr-global	–	–	

*MNI* MNI stereotactic coordinates, *p*_*FWE*_ family-wise error corrected *p*-value.

**Table 2:** Brain activation areas significantly associated with each covariate (verbal hallucination proneness, visual imagery, sex, verbal IQ, and Pr-global) during the false recognition of high- and low-frequency words in the 37 participants.

Contrasts	Covariates	Cluster size and significance (p_FWE_)	Cluster peak coordinates* (MNI)	Region
False recognition of high-frequency words	Verbal hallucination	–	–	
	Visual imagery	Neg. 143 (0.007)	− 3, − 34, 35	Posterior cingulate
		Neg. 108 (0.02)	− 57, − 43, − 10	Left planum temporale
		**Neg. 94 (0.032)**	− **45, 26, 17**	Broca’s area
		Neg. 82 (0.049)	24, − 73, − 37	Right cerebellum (crus 1)
	Sex	–	–	
	Verbal IQ	–	–	
	Pr-global	–	–	
False recognition of low-frequency words	Verbal hallucination	Pos. 182 (0.001)	− 39, − 28, 8	Left Heschl +
			− 42, 8, 11	Broca’s area
		**Pos. 82 (.033)**	**0, 35, 17**	Anterior cingulate
	Visual imagery	**–**	**–**	
	Sex	**–**	**–**	
	Verbal IQ	–	–	
	Pr-global	Neg. 75 (0.044)	− 18, − 31, 65 − 6, − 34, 53	Left post-central gyrus + para central lobule

*MNI *MNI stereotactic coordinates, *p*_*FWE*_ family-wise error corrected *p*-value, *Peak-level corrected significance *p*_FWE_ < .05 marked in bold coordinates.

When the LSHS score was entered in the model instead of the verbal hallucination score, the analyses did not reveal any significant activation associated with this global hallucination score during the correct or false recognitions of either high- or low-frequency words. When the delusion proneness score was entered in the model instead of the verbal or global hallucination score, delusion proneness was found to be significantly associated with increased activation of various brain areas—right cerebellum, left middle frontal gyrus, bilateral middle temporal gyrus—during the correct recognitions of low-frequency words and the false recognitions of high-frequency words (see Table 3).

**Table 3:** Brain activation areas significantly associated with delusion proneness during the correct and false recognition of words, in the 37 participants.

Contrasts	Cluster size and significance (p_FWE_)	Cluster peak coordinates* (MNI)	Region
Correct recognition of low-frequency words	Pos. 86 (.023)	18, − 70, − 22	Right cerebellum (crus VI)
	Pos. 85 (.024)	− 33, 5, 50	Left middle frontal gyrus
False recognition high-frequency words	Pos. 202 (0.001)	− 51, − 61, 2	Left middle temporal gyrus
	Pos. 188 (0.002)	24, − 76, − 28	Right cerebellum (crus I)
	Pos. 108 (0.023)	45, − 61, − 1	Right middle temporal gyrus
	**Pos. 92 (.039)**	− **30, 5, 56**	Left middle frontal gyrus

The regression analysis model includes delusion proneness, visual imagery, sex, verbal IQ, and Pr-global as predictors.

*MNI* MNI stereotactic coordinates, *p*_*FWE*_ family-wise error corrected *p*-value, *Peak-level corrected significance *p*_FWE_ < .05 marked in bold coordinates.

## Discussion

### Verbal hallucination proneness

Verbal hallucinations are the most commonly observed type of hallucination in patients with schizophrenia, and they are assumed to stem from self-monitoring failure through which inner speech is misattributed to an external source^48–52^. Inner speech has also been linked to auditory-verbal hallucination proneness in non-clinical individuals^53–55^. One factor potentially relevant to the study of inner speech is the sense of familiarity conveyed by the verbal material that is being processed, and we therefore varied the frequency of use of the experimental words. As expected, proneness to hallucinations in the verbal modality was significantly associated with increased rates of false recognition of the high-frequency, but not the low-frequency words. This observation extends a finding previously observed in schizophrenia patients to the general population^25^.

The pattern of differential associations with cerebral activity confirms the implication of word familiarity in false recognitions. Verbal hallucination proneness was associated with activation of language and decisional brain regions during the false recognition of non-target words. However, these associations pertained only to the low-frequency words. During the false recognitions of these words, hallucination proneness was indeed associated with significant activation of left Heschl’s gyrus and Broca’s area, both involved in speech production. Cortical regions engaged in language reception and language production are consistently activated during auditory-verbal hallucinations^56^, and left Heschl’s gyrus in particular has been proposed as a key region for this symptom^57,58^. No similar activation was observed for the high-frequency words. One interpretation is that inner speech might be over-salient in verbal hallucination-prone individuals, and therefore the feeling of familiarity conveyed by common words might be so sharp that their processing requires little activation of language-related areas.

Further, verbal hallucination proneness was significantly associated with activation of the anterior cingulate cortex during the false recognition of low-frequency words. The anterior cingulate cortex is involved in decision-making in conflictual situations^59^. In schizophrenia patients it has been found to be involved in the appraising of errors^60,61^. Previous neuroimaging studies have revealed activation of the anterior cingulate during false recognition of faces in healthy participants^62^, and false memories for pictures in healthy participants^63^ and schizophrenia patients^64^. Interestingly, it was reported that schizophrenia patients with verbal hallucinations, in contrast to healthy participants and non-hallucinating patients, failed to activate the anterior cingulate during the appraisal of self/alien speech^35^ and the generation of inner speech^65^. In our verbal hallucination-prone participants, a similar lack of significant activation of this brain region during false recognition of non-target high-frequency words suggests that these words, exceedingly accessible, were confidently sensed as having been recently presented, while the expected cognitive conflict arose for the judgement of the non-target low-frequency words. The anterior cingulate might be crucially involved in the cognitive biases associated with hallucinations. Indeed, a recent review identified this brain structure as a shared neural mechanism of aberrant salience and source monitoring in psychosis.^66^.

The examination of correct recognitions further demonstrates the differential processing of familiar vs. uncommon words in verbal hallucination-prone individuals. Indeed, during correct recognitions of low-frequency words, verbal hallucination proneness was associated with activation of the lingual gyrus, engaged in the visual processing of words^67^, as well as with activation of a brain region involved in memory retrieval, namely the left angular gyrus^68^. No similar activations were observed during correct recognitions of high-frequency words, which suggests that these correct recognitions arose more from a guess than from an authentic retrieval. Thus, the pattern of behavioural and neuroimaging findings indicates that in verbal hallucination-prone individuals, the experimental words that seemed familiar, be they targets or distractors, were liberally endorsed as previously presented —i.e., perceived—words without implementing of the necessary linguistic, recollective, and decisional processes. It is worth noting that the differential pattern of cerebral activity and response bias as a function of word frequency is specific to hallucination proneness in the verbal modality. Indeed, the global hallucination proneness score was significantly associated with liberal response bias for both types of word, and it was not associated with any cerebral activity for either. A technical point should be made that the emergence of a dissociated pattern in verbal hallucination-prone participants is likely to have been facilitated by the experimental procedure of intermixing high- and low-frequency words in the recognition list, thereby increasing their differential processing. Less distinctive results might have been observed if pure recognition lists of high-frequency and of low-frequency words had been contrasted.

Previous cognitive studies that have employed other paradigm types have similarly demonstrated involvement of impaired self-monitoring of inner speech in non-clinical hallucinations^14,29,30,69–71^. Confusion between inner speech and perception appears to be a shared underpinning of clinical and non-clinical verbal hallucinations, supporting a continuum model of verbal hallucinations^72–76^. This confusion may stem from defective self-monitoring comparison processes, impeding appropriate evaluation of internal vs. external production. In our study, though, the fact that decisional processes were adequately implemented for judging the low-frequency words suggests that self-monitoring comparison processes were not intrinsically defective but rather that they failed to be recruited for the judging of the familiar words. Self-monitoring errors might also result from abnormal salience of inner speech through increased vividness or increased abundance of this material. Within the source monitoring framework, abnormal vividness of inner speech would make it seem more similar to perceived speech; abnormal abundance of inner speech, reflecting an easy production without cognitive effort, would make it seem dissimilar to cognitively-produced internal events, and therefore more likely to be mistaken for perception^8,77–79^. Abnormal salience of inner speech might be a characteristic of individuals presenting clinical or non-clinical verbal hallucinations, while a clinical hallucinatory level might be reached when self-monitoring disruption further occurs. It should be kept in mind that cognitive mechanisms other than inner speech misattribution, such as intrusive memories and cognitive disinhibition^5,80^, are also likely to participate in the formation of verbal hallucinations.

### Visual imagery

Meanwhile, visual imagery, which also contributes to psychotic experience, was not associated with increased rates of false recognition of either type of word, and its pattern of associations with cerebral activity was entirely distinct from that observed for verbal hallucination proneness. Visual imagery relies largely on the same cortical bases as visual perception^81^, and it appears to have an impact on verbal processing. Indeed, various fMRI studies which contrasted concrete vs. abstract words or manipulated word imageability have demonstrated that the processing of highly imageable concrete words was associated with activation of visual-related brain regions^82,83^. A study focused on visual imagery revealed that only a subgroup of individuals who demonstrated high visual imagery propensity activated a visual brain region during the processing of common concrete words, suggesting that they had formed a visual mental image of the designated object^43^. In the current study, visual imagery selectively impacted the judgment of high-frequency words, as expected, and it was associated with decreased, rather than increased, cerebral activity. In particular, during false recognitions of high-frequency words, higher visual imagery scores were associated with decreased activation of two regions involved in memory retrieval, the posterior cingulate cortex and the cerebellum-crus 1, and of two verbal areas, namely the left planum temporale and Broca’s area. Individuals with high visual imagery scores probably made visual mental images of the familiar words that were presented, thereby de-activating the brain regions usually recruited for verbal processing. This observation is compatible with studies which used the Deese–Roediger–McDermott paradigm and demonstrated that the instruction to form visual mental images of target words at encoding resulted in fewer false recognitions of non-target words^84–86^.

### Delusion proneness

High levels of delusion proneness did not lead to increased rates of false recognitions of words, corroborating what was observed in another healthy sample^25^. This suggests that misattribution of inner speech is not a mechanism involved in this symptom. Our behavioural data reveal that delusion proneness was associated, rather, with rapidness in making false recognitions of both high and low-frequency words. These short response times might reflect the ‘jumping-to-conclusions’ bias and overconfidence in incorrect memories consistently observed in delusional schizophrenia patients and non-clinical delusion-prone individuals^87–89^. At the cortical level, delusion proneness was associated with activation of the right cerebellum-crus I, a brain region engaged in autobiographical memory retrieval^90^, during false recognition of high-frequency words. It was further associated with bilateral activation of the middle temporal gyrus, which is involved in semantic processing^91,92^. False recognitions in delusion-prone individuals might result from semantic and reasoning abnormalities rather than from any deficiency in the monitoring of self-generated information.

## Limitations and conclusions

Our conclusions are limited by the low incidence of verbal hallucination proneness in the sample and the restricted range observed for this symptom score. The differential processing of high- vs. low-frequency words ought to be tested in a large sample of verbal-hallucination prone individuals. It should be noted, though, that the analyses revealed significant associations of cerebral activation with the verbal but not the global hallucination score, in spite of the much more extended range of this latter score. Another important limitation is that the manipulation of word frequency can only be assumed to tap into the processes engaged in inner speech. At the methodological level, the fact that the word presentation format was different at encoding and at recognition may have to some extent affected the results. Nonetheless, our combined behavioural and neuroimaging data corroborate the view that proneness to verbal hallucinations in non-clinical individuals, similar to verbal hallucinations in schizophrenia patients, hinges on a decreased ability to distinguish inner speech from perceived verbal information. With respect to the other psychosis-related factors that we investigated, visual imagery was associated with deactivation of language-related brain areas during false memories of highly imageable words, while proneness to delusions appears to be associated with hasty decisions rather than with increased rates of false memories.

## Data Availability

The data are available upon request to Drs Gildas Brébion and Christian Stephan-Otto.

## References

[1] Kråkvik B (2015). Prevalence of auditory verbal hallucinations in a general population: A group comparison study. Scand. J. Psychol..

[2] Johns LC (2005). Hallucinations in the general population. Curr. Psychiatry Rep..

[3] Maijer K, Begemann MJH, Palmen SJMC, Leucht S, Sommer IEC (2018). Auditory hallucinations across the lifespan: A systematic review and meta-analysis. Psychol. Med..

[4] Johns LC (2014). Auditory verbal hallucinations in persons with and without a need for care. Schizophr. Bull..

[5] Badcock JC, Hugdahl K (2012). Cognitive mechanisms of auditory verbal hallucinations in psychotic and non-psychotic groups. Neurosci. Biobehav. Rev..

[6] Waters FAV (2012). Auditory hallucinations in schizophrenia and nonschizophrenia populations: A review and integrated model of cognitive mechanisms. Schizophr. Bull..

[7] de Leede-Smith S, Barkus E (2013). A comprehensive review of auditory verbal hallucinations: lifetime prevalence, correlates and mechanisms in healthy and clinical individuals. Front. Hum. Neurosci..

[8] Johnson MK, Hashtroudi S, Lindsay DS (1993). Source monitoring. Psychol. Bull..

[9] Brookwell ML, Bentall RP, Varese F (2013). Externalizing biases and hallucinations in source-monitoring, self-monitoring and signal detection studies: A meta-analytic review. Psychol. Med..

[10] Larøi F, Woodward TS (2007). Hallucinations from a cognitive perspective. Harv. Rev. Psychiatry.

[11] Bentall RP, Slade PD (1985). Reality testing and auditory hallucinations: A signal detection analysis. Br. J. Clin. Psychol..

[12] Vercammen A, de Haan EHF, Aleman A (2008). Hearing a voice in the noise: Auditory hallucinations and speech perception. Psychol. Med..

[13] Varese F, Barkus E, Bentall RP (2012). Dissociation mediates the relationship between childhood trauma and hallucination-proneness. Psychol. Med..

[14] Rankin PM, O’Carroll PJ (1995). Reality discrimination, reality monitoring and disposition towards hallucination. Br. J. Clin. Psychol..

[15] Barkus E, Stirling J, Hopkins R, McKie S, Lewis S (2007). Cognitive and neural processes in non-clinical auditory hallucinations. Br. J. Psychiatry.

[16] Varese F, Barkus E, Bentall RP (2011). Dissociative and metacognitive factors in hallucination-proneness when controlling for comorbid symptoms. Cognit. Neuropsychiatry.

[17] Smailes D, Meins E, Fernyhough C (2015). Associations between intrusive thoughts, reality discrimination and hallucination-proneness in healthy young adults. Cognit. Neuropsychiatry.

[18] Alganami F, Varese F, Wagstaff GF, Bentall RP (2017). Suggestibility and signal detection performance in hallucination-prone students. Cognit. Neuropsychiatry.

[19] Moseley P (2022). Continuities and discontinuities in the cognitive mechanisms associated with clinical and nonclinical auditory verbal hallucinations. Clin. Psychol. Sci..

[20] Moseley P, Smailes D, Ellison A, Fernyhough C (2016). The effect of auditory verbal imagery on signal detection in hallucination-prone individuals. Cognition.

[21] de Boer JN (2019). Auditory hallucinations, top-down processing and language perception: A general population study. Psychol. Med..

[22] Laloyaux J, Hirnstein M, Specht K, Giersch A, Larøi F (2022). Eliciting false auditory perceptions using speech frequencies and semantic priming: A signal detection approach. Cognit. Neuropsychiatry.

[23] Brébion G, Smith MJ, Amador X, Malaspina D, Gorman JM (1998). Word recognition, discrimination accuracy, and decision bias in schizophrenia: Association with positive symptomatology and depressive symptomatology. J. Nerv. Ment. Dis..

[24] Brébion G, David AS, Jones H, Pilowsky LS (2005). Hallucinations, negative symptoms, and response bias in a verbal recognition task in schizophrenia. Neuropsychology.

[25] Brébion G (2016). Impaired self-monitoring of inner speech in schizophrenia patients with verbal hallucinations and in non-clinical individuals prone to hallucinations. Front. Psychol..

[26] Brébion G, David AS, Ohlsen R, Jones HM, Pilowsky LS (2007). Visual memory errors in schizophrenic patients with auditory and visual hallucinations. J. Int. Neuropsychol. Soc..

[27] Brébion G (2019). Clinical and non-clinical hallucinations are similarly associated with source memory errors in a visual memory task. Conscious. Cogn..

[28] Brébion G, Larøi F, Van Der Linden M (2010). Associations of hallucination proneness with free-recall intrusions and response bias in a nonclinical sample. J. Clin. Exp. Neuropsychol..

[29] Sugimori E, Asai T, Tanno Y (2011). Sense of agency over thought: External misattribution of thought in a memory task and proneness to auditory hallucination. Conscious. Cogn..

[30] Kanemoto M, Asai T, Sugimori E, Tanno Y (2013). External misattribution of internal thoughts and proneness to auditory hallucinations: The effect of emotional valence in the Deese–Roediger–McDermott paradigm. Front. Hum. Neurosci..

[31] Richards SE, Hughes ME, Woodward TS, Rossell SL, Carruthers SP (2021). External speech processing and auditory verbal hallucinations: A systematic review of functional neuroimaging studies. Neurosci. Biobehav. Rev..

[32] Scheinost D, Tokoglu F, Hampson M, Hoffman R, Constable RT (2019). Data-driven analysis of functional connectivity reveals a potential auditory verbal hallucination network. Schizophr. Bull..

[33] Altamura M (2020). Do patients with hallucinations imagine speech right?. Neuropsychologia.

[34] Mitchell KJ, Johnson MK (2009). Source monitoring 15 years later: What have we learned from fMRI about the neural mechanisms of source memory?. Psychol. Bull..

[35] Allen P (2007). Neural correlates of the misattribution of speech in schizophrenia. Br. J. Psychiatry.

[36] Kumari V (2010). Functional MRI of verbal self-monitoring in schizophrenia: Performance and illness-specific effects. Schizophr. Bull..

[37] Simons JS, Garrison JR, Johnson MK (2017). Brain mechanisms of reality monitoring. Trends Cognit. Sci..

[38] Dennis NA, Bowman CR, Turney IC, Addis DR, Barense M, Duarte A (2015). Functional neuroimaging of false memories. The Wiley Handbook on the Cognitive Neuroscience of Memory.

[39] Schuster S, Hawelka S, Hutzler F, Kronbichler M, Richlan F (2016). Words in context: The effects of length, frequency, and predictability on brain responses during natural reading. Cereb. Cortex.

[40] Gomar JJ (2011). Validation of the Word Accentuation Test (TAP) as a means of estimating premorbid IQ in Spanish speakers. Schizophr. Res..

[41] Launay G, Slade P (1981). The measurement of hallucinatory predisposition in male and female prisoners. Pers. Individ. Dif..

[42] Siddi S (2018). Measurement invariance of the Spanish Launay-Slade hallucinations scale-extended version between putatively healthy controls and people diagnosed with a mental disorder. Int. J. Methods Psychiatr. Res..

[43] Stephan-Otto C (2017). Visual imagery and false memory for pictures: A functional magnetic resonance imaging study in healthy participants. PLoS ONE.

[44] Peters E, Joseph S, Day S, Garety P (2004). Measuring delusional ideation: The 21-item Peters et al. Delusions inventory (PDI). Schizophr. Bull..

[45] Corwin J (1994). On measuring discrimination and response bias: Unequal numbers of targets and distractors and two classes of distractors. Neuropsychology.

[46] Bolla-Wilson K, Bleecker ML (1986). Influence of verbal intelligence, sex, age, and education on the rey auditory verbal learning test. Dev. Neuropsychol..

[47] Mortensen EL, Gade A (1993). On the relation between demographic variables and neuropsychological test performance. Scand. J. Psychol..

[48] Alderson-Day B, Fernyhough C (2015). Inner speech: Development, cognitive functions, phenomenology, and neurobiology. Psychol. Bull..

[49] Allen P, Aleman A, McGuire PK (2007). Inner speech models of auditory verbal hallucinations: Evidence from behavioural and neuroimaging studies. Int. Rev. Psychiatry.

[50] Moseley P, Fernyhough C, Ellison A (2013). Auditory verbal hallucinations as atypical inner speech monitoring, and the potential of neurostimulation as a treatment option. Neurosci. Biobehav. Rev..

[51] Rosen C (2018). The tangled roots of inner speech, voices and delusions. Psychiatry Res..

[52] Stephane M (2019). The self, agency and spatial externalizations of inner verbal thoughts, and auditory verbal hallucinations. Front. Psychiatry.

[53] McCarthy-Jones S, Fernyhough C (2011). The varieties of inner speech: Links between quality of inner speech and psychopathological variables in a sample of young adults. Conscious. Cognit..

[54] Alderson-Day B (2014). Shot through with voices: Dissociation mediates the relationship between varieties of inner speech and auditory hallucination proneness. Conscious. Cognit..

[55] Alderson-Day B, Mitrenga K, Wilkinson S, McCarthy-Jones S, Fernyhough C (2018). The varieties of inner speech questionnaire—revised (VISQ-R): Replicating and refining links between inner speech and psychopathology. Conscious. Cognit..

[56] Zmigrod L, Garrison JR, Carr J, Simons JS (2016). The neural mechanisms of hallucinations: A quantitative meta-analysis of neuroimaging studies. Neurosci. Biobehav. Rev..

[57] Modinos G (2013). Neuroanatomy of auditory verbal hallucinations in schizophrenia: A quantitative meta-analysis of voxel-based morphometry studies. Cortex.

[58] Shinn AK, Baker JT, Cohen BM, Öngür D (2013). Functional connectivity of left Heschl’s gyrus in vulnerability to auditory hallucinations in schizophrenia. Schizophr. Res..

[59] Botvinick MM, Braver TS, Barch DM, Carter CS, Cohen JD (2001). Conflict monitoring and cognitive control. Psychol. Rev..

[60] Polli FE (2008). Reduced error-related activation in two anterior cingulate circuits is related to impaired performance in schizophrenia. Brain.

[61] Kerns JG (2005). Decreased conflict- and error-related activity in the anterior cingulate cortex in subjects with schizophrenia. Am. J. Psychiatry.

[62] Iidaka T, Harada T, Kawaguchi J, Sadato N (2012). Neuroanatomical substrates involved in true and false memories for face. Neuroimage.

[63] Okado Y, Stark C (2003). Neural processing associated with true and false memory retrieval. Cognit. Affect. Behav. Neurosci..

[64] Stephan-Otto C (2017). Remembering verbally-presented items as pictures: Brain activity underlying visual mental images in schizophrenia patients with visual hallucinations. Cortex.

[65] Simons CJP (2010). Functional magnetic resonance imaging of inner speech in schizophrenia. Biol. Psychiatry.

[66] Kowalski J, Aleksandrowicz A, Dąbkowska M, Gawęda Ł (2021). Neural correlates of aberrant salience and source monitoring in schizophrenia and at-risk mental states—a systematic review of fmri studies. J. Clin. Med..

[67] Mechelli A, Humphreys GW, Mayall K, Olson A, Price CJ (2000). Differential effects of word length and visual contrast in the fusiform and lingual gyri during reading. Proc. Biol. Sci..

[68] Rugg MD, King DR (2018). Ventral lateral parietal cortex and episodic memory retrieval. Cortex.

[69] Larøi F, Van der Linden M, Marczewski P (2004). The effects of emotional salience, cognitive effort and meta-cognitive beliefs on a reality monitoring task in hallucination-prone subjects. Br. J. Clin. Psychol..

[70] Alderson-Day B (2017). Distinct processing of ambiguous speech in people with non-clinical auditory verbal hallucinations. Brain.

[71] Gupta T, DeVylder JE, Auerbach RP, Schiffman J, Mittal VA (2018). Speech illusions and working memory performance in non-clinical psychosis. Schizophr. Res..

[72] Baumeister D, Sedgwick O, Howes O, Peters E (2017). Auditory verbal hallucinations and continuum models of psychosis: A systematic review of the healthy voice-hearer literature. Clin. Psychol. Rev..

[73] Pinheiro AP, Schwartze M, Kotz SA (2018). Voice-selective prediction alterations in nonclinical voice hearers. Sci. Rep..

[74] Powers AR, van Dyck LI, Garrison JR, Corlett PR (2020). Paracingulate sulcus length is shorter in voice-hearers regardless of need for care. Schizophr. Bull..

[75] Pinheiro AP, Schwartze M, Kotz SA (2020). Cerebellar circuitry and auditory verbal hallucinations: An integrative synthesis and perspective. Neurosci. Biobehav. Rev..

[76] Castiajo P, Pinheiro AP (2021). Acoustic salience in emotional voice perception and its relationship with hallucination proneness. Cognit. Affect. Behav. Neurosci..

[77] Sugimori E, Mitchell KJ, Raye CL, Greene EJ, Johnson MK (2014). Brain mechanisms underlying reality monitoring for heard and imagined words. Psychol. Sci..

[78] Fazekas P (2021). Hallucinations as intensified forms of mind-wandering. Philos. Trans. R. Soc. B Biol. Sci..

[79] Dijkstra N, Kok P, Fleming SM (2022). Perceptual reality monitoring: Neural mechanisms dissociating imagination from reality. Neurosci. Biobehav. Rev..

[80] Allé MC, Berna F, Berntsen D (2018). Involuntary autobiographical memory and future thought predicting hallucination proneness. Clin. Psychol. Sci..

[81] Dijkstra N, Bosch SE, van Gerven MAJ (2019). Shared neural mechanisms of visual perception and imagery. Trends Cognit. Sci..

[82] Mestres-Missé A, Münte TF, Rodriguez-Fornells A (2009). Functional neuroanatomy of contextual acquisition of concrete and abstract words. J. Cognit. Neurosci..

[83] Garbarini F (2020). Imageability effect on the functional brain activity during a naming to definition task. Neuropsychologia.

[84] Robin F, Ménétrier E, Beffara Bret B (2021). Effects of visual imagery on false memories in DRM and misinformation paradigms. Memory.

[85] Robin F, Mahé A (2015). Effects of image and verbal generation on false memory. Imag. Cognit. Pers..

[86] Foley MA (2012). Imagery encoding and false recognition errors: Exploring boundary conditions of imagery’s enhancing effects. Memory.

[87] Garety PA, Freeman D (2013). The past and future of delusions research: From the inexplicable to the treatable. Br. J. Psychiatry.

[88] Andreou C, Moritz S, Veith K, Veckenstedt R, Naber D (2014). Dopaminergic modulation of probabilistic reasoning and overconfidence in errors: A double-blind study. Schizophr. Bull..

[89] Moritz S, Woodward TS (2006). Metacognitive control over false memories: A key determinant of delusional thinking. Curr. Psychiatry Rep..

[90] Addis DR, Moloney EEJ, Tippett LJ, Roberts RP, Hach S (2016). Characterizing cerebellar activity during autobiographical memory retrieval: ALE and functional connectivity investigations. Neuropsychologia.

[91] Jefferies E (2013). The neural basis of semantic cognition: Converging evidence from neuropsychology, neuroimaging and TMS. Cortex.

[92] Python G, Glize B, Laganaro M (2018). The involvement of left inferior frontal and middle temporal cortices in word production unveiled by greater facilitation effects following brain damage. Neuropsychologia.

